# Evolving growth hormone deficiency: proof of concept

**DOI:** 10.3389/fendo.2024.1398171

**Published:** 2024-05-01

**Authors:** Sri Nikhita Chimatapu, Swathi Sethuram, Julie G. Samuels, Alexandra Klomhaus, Cassie Mintz, Martin O. Savage, Robert Rapaport

**Affiliations:** ^1^ Division of Pediatric Endocrinology, University of California, Los Angeles (UCLA) Mattel Children’s Hospital, Los Angeles, CA, United States; ^2^ Division of Pediatric Endocrinology, Massachusetts General Hospital, Boston, MA, United States; ^3^ Division of Pediatric Endocrinology and Diabetes, Icahn School of Medicine at Mount Sinai, New York, NY, United States; ^4^ Department of Medicine Statistics Core, David Geffen School of Medicine, University of California Los Angeles, Los Angeles, CA, United States; ^5^ Division of Medical Genetics & Genomics, Department of Genetics & Genomic Sciences, Icahn School of Medicine at Mount Sinai, New York, NY, United States; ^6^ William Harvey Research Institute, Barts and the London School of Medicine & Dentistry, University of London, Queen Mary, United Kingdom

**Keywords:** growth, growth hormone stimulation test, growth hormone deficiency, idiopathic short stature, growth hormone therapy

## Abstract

**Introduction:**

We present the evolution of GHD in adolescent males with persistent growth failure, in whom the diagnosis was established after a second GH stimulation test (GST).

**Methods:**

We performed a retrospective chart review of children who presented for short stature (height less < 2SD for mean/mid-parental height) and/or growth failure (sustained growth velocity < 0 SD) to pediatric endocrinology at Mount Sinai Kravis Children’s Hospital, New York and who had 2 GSTs. Data collected from electronic medical records were analyzed using SPSS v28.0

**Results:**

Of 53 patients included, 42 were males. Average GH peak on initial GST was 15.48 ± 4.92 ng/ml, at 10.07 ± 2.65 years, mean height -1.68 ± 0.56SD(28% had <2SD), IGF-1 -1.00 ± 0.88SD. After 2.23 ± 1.22 years, at 12.04 ± 2.41years, height SDs decreased to -1.82 ± 0.63SD and IGF-1 was -1.08 ± 0.84SD. At repeat GST, average GH peak was 7.59 ± 2.12 ng/dL, with 36% ≤7 ng/dl and 32% in puberty. 12 males reached adult height of 0.08 ± 0.69 SD with a mean height gain of 1.83 ± 0.56SD(p<0.005), IGF-1 of -1.15 ± 0.81SD after 4.64 ± 1.4 years of GH.

**Conclusion:**

We offer evidence for Evolving Growth Hormone Deficiency (EGHD) through repeat GST in children with persistent growth slowdown, even with pubertal progression; emphasizing the need for careful longitudinal follow-up to make accurate diagnosis.

## Introduction

Growth hormone deficiency (GHD) is a common endocrinological cause of growth failure and short stature with a reported incidence of 1/4,000 to 1/10,000 children (1). GHD can be congenital or acquired and be present in combination with other pituitary deficiencies or as an isolated defect. The diagnosis of isolated, acquired GHD (IGHD), which represents the majority of cases, can be challenging (2). The GH Research Society consensus statement recommends that IGHD diagnosis should include auxological, biochemical, and radiographic evaluations (1, 3–5). In children with clinical criteria for GHD, growth hormone stimulation tests (GST) continue to play a key role in the diagnosis despite the known limitations of GST (6–9). A peak growth hormone (GH) level below 10 µg/dL is largely still clinically considered the cut-off for the diagnosis of GHD (9–11) in the United States and in other countries across the globe (12). Recent multi center international clinical studies regarding long-acting GH also all utilized 10mcg/dl as the GHD cut off (13, 14).

Children with a height more than 2.25SDs below the mean with no identified cause for short stature following a thorough history, physical exam, screening evaluations and GST are labelled as “idiopathic short stature” (ISS) or short stature of unknown etiology (10, 15). As children undergo more extensive evaluations, researchers proposed that the diagnosis of ISS should only be considered after detailed genetic evaluations and thorough reevaluation, including retesting with GST (16, 17).

Patients with organic brain lesions or those who have undergone cranial irradiation have been described as having GHD that evolves over time following the initial evaluation (18–22). We recently reported two patients who upon careful longitudinal monitoring and retesting of the growth hormone axis, had a diagnosis consistent with evolving IGHD without an organic brain lesion, similar to three patients previously reported by Zadik et al. (18–23). We postulate that even in patients without organic lesions careful longitudinal monitoring and retesting with GST will identify patients with findings consistent with the diagnosis of IGHD who may benefit from treatment with GH. We propose the term evolving growth hormone deficiency (EGHD) for these patients. Currently, the prevalence of this entity is unknown.

We investigated the clinical and biochemical characteristics of a cohort of 56 children followed at a single center for short stature or growth failure who had two or more GSTs due to continued suboptimal growth. We identified the characteristics of children who may be at risk for developing EGHD and evaluated their response to recombinant human GH therapy. We present adult height data in 12 of the 25 the GH treated patients with EGHD. Our findings emphasize the importance of re-evaluating children with continued evidence of inadequate growth despite a previously normal GST and who may otherwise be prematurely, and inaccurately labeled as having ‘ISS’

## Methods

The retrospective study was approved by the Institutional Review Board at the Icahn School of Medicine at Mount Sinai. We reviewed medical records of children who were referred to the division of Pediatric Endocrinology and Diabetes at Mount Sinai for concerns of growth (ICD-10) diagnosis code R62.50) between January 2015 and December 2020 and who underwent more than one GST as part of their evaluation. Short stature was defined as height more than 2 standard deviations (SD) below the mean for the population or for the family, using the Hermanussen and Cole definition of target height (24). And defined growth failure as patients presenting with sustained growth velocity < 0SD. Children who did not undergo more than one Growth Hormone Stimulation Test (GST), those with severe chronic illnesses (such as Inflammatory Bowel Disease, Celiac Disease, or others), or those with known genetic syndromes associated with poor growth (such as Turner syndrome, Noonan syndrome, or SHOX deficiency), as well as other identifiable causes of poor growth, were excluded from the study. None of our participants was on medications known to interfere with growth. None had received previous treatment with growth hormone. *Data Collection:* We collected clinical data including age, sex, height, and weight. BMI was calculated as weight (kg) divided by height (m) squared. Reliable height measurements were obtained by a pediatric endocrinologist using Holtain stadiometer that was calibrated weekly. Heights were plotted on the Center for Disease Control and Prevention growth chart. Height SDS and growth velocities were determined using CDC growth charts on electronic medical records. Pubertal stage (25) was obtained from the documented physical examination by the same pediatric endocrinologist and evaluated biochemically at the Endocrine Sciences laboratory. Pubertal thresholds were set at the following values: Tanner 2 breast development on examination with baseline morning LH ≥ 0.3 IU/L and baseline morning estradiol ≥ 20 pg/mL (37 pmol/L) in girls or testicular volumes ≥ 4ml with LH ≥ 0.3 IU/L and testosterone > 20ng/dL in boys (26).

Serum concentrations of IGF-1, IGFBP-3, follicle-stimulating hormone (FSH), luteinizing hormone (LH), testosterone, estradiol, FreeT4, Thyroid stimulating hormone and cortisol, DHEA-Sulfate were obtained before each GST from Endocrine Sciences via liquid chromatography with tandem mass spectrometry(LC/MS-MS), and during follow up visits when on GH therapy. IGF-1 Z scores were calculated according to chronological age, sex and puberty.IGF-1 Z scores were calculated according to chronological age, sex and puberty; we used the LabCorp IGF-I Z-score calculator for reference. We also collected bone ages (BA) determined by pediatric radiologists as well as endocrinologists according to the standards established by Greulich and Pyle (27) based on radiographs of the left hand and wrist.

All GSTs were performed at 08.00 am following a minimum 8-hour overnight fast. Patients received a combination of two provocative agents simultaneously the same day: 10% arginine HCL (0.5 g/kg) and oral L-dopa dose (10 mg/kg max dose of 500 mg) or IM glucagon (30 µg/kg) at time 0. Blood samples for serum GH concentrations were obtained at baseline and 30, 60, 90, 120, 150 and 180 minutes after the administration of firstagent. Serum GH was measured by the double-antibody RIA method by Endocrine Sciences Laboratory. This assay uses reagent antibodies that are polyclonal and detects 22kDA (primary GH isoform) plus other GH isoforms. This test is calibrated against International Reference Preparation (IRP) international standard (IS) 80/505 of human pituitary origin. This contains all GH isoforms. The intra- and inter-imprecision coefficient of variation are <10%.

Repeat GSTs were performed on patients who had a further decrease in growth velocity and height SDS, despite advancing puberty. Males and females were divided into GH deficient (GHD), and GH sufficient (GHS) groups based on their response to GST(Patients with peak GH of ≥ 10ng/mL were labeled as GHS and those with peak GH <10 ng/mL were labeled as the GHD cohort. Participants in the GHD cohort had Magnetic Resonance imaging (MRI) of their brain before the initiation of GH therapy evaluated by the same neuroradiologist All patients with GHD were offered GH therapy and were treated with recombinant human GH therapy (rhGH) at an initial dose ranging from 0.18 to 0.28 mg/kg/week.

### Statistical analysis

We present descriptive statistics, including means and standard deviations for continuous variables, and frequencies for categorical variables. We considered a two-sided *p-value* (alpha level) threshold of ≤0.05 as statistically significant, and > 0.05 as not significant (ns). Female subjects were excluded after baseline evaluation because of the small sample size, and all subsequent analyses were performed on males only.

For continuous variables, Wilcoxon rank-sum tests (e.g., two-sample Wilcoxon tests) were used to compare treatment outcomes between GHD and GHS groups. Wilcoxon signed-rank tests (e.g., one-sample/paired-sample Wilcoxon tests) were used to compare outcomes within each GHD and GHS group to evaluate changes in growth and biochemical parameters at various time points during longitudinal follow-up. For categorical variables, chi-square tests (and Fisher’s exact tests, when appropriate) were used to compare between groups, and McNemar’s tests were used to compare longitudinal frequencies within groups. Data were analyzed using SPSS version 28 and SAS 9.4 (SAS Institute, Cary NC).

## Results

Fifty-six patient charts were reviewed and 53 met all the inclusion criteria. Of the 53 children that were included, 42 were males (9.33 ± 2.58 years of age) and 11 were females (8.19 ± 2.76 years of age) at initial GST (**Table 1**). None of our patients was overweight or obese; BMI was normal at baseline as well as the end of GH treatment. Of the 42 males retested with a GST after 2.1+/- 1.22 years, 25 had GHD whereas 17 were GHS. Those who had GHD, underwent MRI of the pituitary which revealed that 10 patients (33.3%) had a small pituitary; the rest were normal. There was no difference in peak GH response between those with a normal MRI versus those with small pituitary glands. Three boys were noted to be in puberty at initial GST (testicular volume >4ml and testosterone> 20 ng/dL). All the patients had puberty at a normal age. There were no outliers with respect to onset of puberty.

**Table 1:** Baseline patient characteristics at initial growth hormone stimulation test (GST).

Characteristics	MALES (N=42)	FEMALES (N=11)	p-value^a^
	Mean ± SD	Mean ± SD	
Age in years	9.33 ± 2.58	8.19 ± 2.76	0.29
Height Z-score	-1.76 ± 0.47	-2.01 ± 0.28	0.24
BMI Z-score	-0.48 ± 0.92	-0.59 ± 1.24	0.58
IGF-1 Z-score	-0.99 ± 0.79	-0.22 ± 0.74	**0.03**
Initial GH peak (ng/ml)	15.98 ± 5.34	16.70 ± 6.22	0.94

a p-value from Wilcoxon two-sample tests. Bolded P-values indicate statistical significance.

**Table 1** shows baseline characteristics for the cohort at the initial GST. The majority of baseline characteristics between males and females were not statistically different. IGF-1 Z score on the contrary was significantly lower in the males (-0.99 ± 0.79 vs -0.22 ± 0.74, p= 0.02).

**Figure 1:**
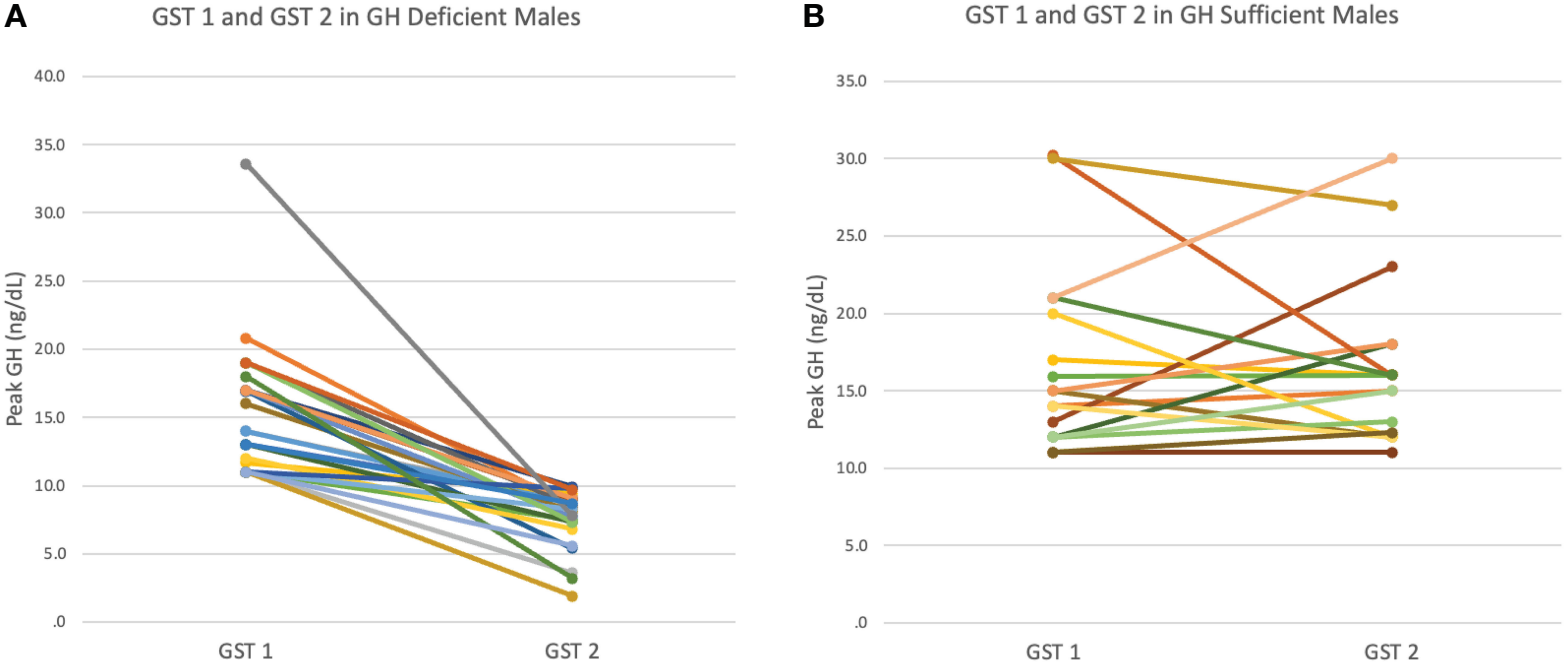
**(A)** Peak GH response at first (GST1) and second (GST2) growth hormone stimulation test in GH Deficient (GHD) males. **(B)** Peak GH response at first (GST1) and second(GST2) growth hormone stimulation test in GH Sufficient (GHS) males.

Male patients’ biochemical and growth data were then compared between their initial and subsequent GSTs (**Table 2**), stratified by those who became GHD on repeat GST and those who remained GHS on repeat GST. There were no significant differences between the ages, mean height Z-scores and mean IGF-1 Z-scores at initial or repeat GST between the GHD and GHS groups. GHD males showed a significantly slower growth velocity (GV) of 2.98 ± 1.32 cm/yr compared to GHS males with a mean GV of 4.932 ± 1.20 cm/yr (p<0.01). There was a decrease in mean height SD in the GHD groups between GST’s, decreasing from -1.68 ± 0.56SD to -1.82 ± 0.63SD, although this did not reach statistical significance, with 28% being < 2 SD at second test. In the GHS group, the height SD remained similar between GST’s, from -1.88 ± 0.28SD at initial GST to -1.85 ± 0.35SD at repeat GST. For both GHD and GHS males, repeat GST was performed on average approximately 2.1 years after initial GST (**Figures 1A, B**).

**Table 2:** Comparison between GST 1 and GST 2 in GHD and GHS males.

Characteristics	GHD (N=25)			GHS (N=17)			GHD vs. GHS at GST1	GHD vs. GHS at Repeat GST
	GST 1	Repeat GST	p-value^a^	GST 1	Repeat GST	p-value	p-value^b^	p-value^c^
	Mean ± SD	Mean ± SD		Mean ± SD	Mean ± SD			
Age in years	10.07 ± 2.65	12.04 ± 2.41	**<.0001**	10.56 ± 1.81	12.44 ± 1.82	**<.0001**	0.51	0.67
Height Z-score	-1.68 ± 0.56	-1.82 ± 0.63	0.21	-1.88 ± 0.28	-1.85 ± 0.35	0.97	0.14	0.45
Testicular volume > 4ml, N (%)	5	8	0.083	3	10	**0.0082**	1.00	0.13
Testosterone in pubertal boys (N=8) (ng/dL)	76.75 ± 152.87	218.71 ± 281.21	**0.03**	7.90 ± 5.85	115.11 ± 139.50	0.06	0.43	0.47
IGF-1 Z-score	-1.05 ± 0.75	-1.08 ± 0.84	0.89	-0.90 ± 0.85	-1.09± 0.99	0.62	0.64	0.99
Growth hormone peak (ng/ml)	15.48 ± 4.92	7.59 ± 2.12	**<.0001**	16.71 ± 5.98	16.61 ± 5.39	0.93	0.54	**<.0001**

a p-value from Wilcoxon paired-sample test comparing GST1 to Repeat GST within groups for continuous variables, and McNemar’s test for categorical variables.

b p-value from Wilcoxon two-sample test comparison of GHD and GHS at GST 1 for continuous variables, and chi-square test for categorical variables.

c p-value from Wilcoxon two-sample test comparison of GHD and GHS at Repeat GST for continuous variables, and chi-square test for categorical variables.

d Do not have information on growth velocity SDS or IGF-1 z-score at repeat GST. Bolded P-values indicate statistical significance.

Of the 25 GHD males, 23 were on treatment with rhGH, one patient did not start therapy during the study period. Data for GHD males were then analyzed at multiple time points after initiating rhGH treatment. 1 year after the start of treatment and at the most recent consultation visit, which was on average 2.56 ± 3.17 years after the repeat GST (**Table 3**). Height Z-score improved by 1.18SD during these years of GH therapy (p<0.001). GV and growth velocity standard deviation score (GVSDS) both showed significant increases at the 1-year treatment mark and at the most recent visit, 9 ± 1.54 cm/year (p<0.001) and 5.01 ± 3.13 cm/year (p<0.005), respectively. IGF-1 Z-score also showed a significant improvement (from -1.08 from the repeat GST to 1.37 at the most recent visit).

**Table 3:** Patient characteristics for boys with GHD at different time points (n=24).

Characteristic	Repeat GST	1 year after GH	p-value^a^	Most recent visit	p-value^b^
	Mean ± SD	Mean ± SD		Mean ± SD	
Height Z-score	-1.82 ± 0.63	-1.37 ± 0.58	**<.0001**	-0.64 ± 1.08	**<.0001**
Height SD gain	n/a	0.55 ± 0.22	n/a	1.21 ± 0.99	0.06
Growth velocity (cm/year)	2.98 ± 1.32	9.00 ± 1.54	**<.0001**	5.01± 3.13	**0.005**
Growth velocity SDS	-1.19 ± 1.51	N/A^c^	N/A	0.82 ± 1.66	**0.002**
IGF-1 Z-score	-1.08 ± 0.84	N/A	N/A	1.36 ± 1.67	**<.0001**

^*^Height values in this table calculated as change from height right before GH stim.

a p-value from Wilcoxon paired-sample test comparing repeat GST to 1 year after GH.

b p-value from Wilcoxon paired-sample test comparing most recent visit to repeat GST.

c Do not have information on growth velocity SDS or IGF-1 z-score at 1 year after GH.

p-value^a^: Statistical significance between Repeat GST and 1 year after GH therapy.

p-value^b^: Statistical significance between Repeat GST and most recent visit.

Time since most recent visit is 2.56 ± 3.17 years. Bolded P-values indicate statistical significance.

At the time of the repeat GST, 8 males out of 25 (32%) in the GHD group were in puberty (mean testicular volume (TV) 9.33 ± 6.0 mL). Despite entering into puberty, they continued to have poor growth velocity of 3.34 ± 1.46 cm/year (p<0.05) and Height Z score -1.46 ± 0.75 SD (**Supplementary Table 1**). On GH treatment, the Height Z score in this cohort had dramatically improved to 0.48 ± 0.27 SDs (p= 0.0625) within one year of start of treatment and 0.04 ± 1.59 SDs (p<0.05) at the most recent visit (2.20 ± 1.80) SDS. Growth velocity improved to 8.84 ± 2.43 cm/year at one year of treatment and 4.33 ± 3.13 cm/year at most recent visit (**Supplementary Table 1**). The difference between bone age and chronological age changed from -1.00 ± 1.04 years to -1.96 ± 1.27 years at the most recent visit (p< 0.05). Further, the IGF-1 Z score had also improved from -1.27 ± 1.19 to 0.92 ± 2.18 SDS (p=0.016). Seventeen males (68%) had not yet entered puberty (mean TV 3.00 ± 1.51) with an average repeat GST of 7.91 ± 1.92 ng/mL. There were no significant differences between mean GST GH peak, height SDS, GV, GVSD or IGF-1 Z-score at repeat GST between pubertal and non-pubertal males (**Supplementary Table 1**). Although not statistically significant, these parameters improved with GH therapy in both the pubertal and non-pubertal groups.

Twelve GHD males treated with rhGH reached adult height (AH).They were treated on average for 4.64 ± 1.36 years. They achieved an average adult height Z-score of 0.08 ± 0.69, an average adult height of 176.53 ± 5.33 cm (**Table 4**). Both, adult height Z-score and adult height were statistically significant when comparing adult height to height at repeat GST(p=0.0005). With GH treatment, the average gain in height SD was 1.85 ± 0.55SD (p<0.001). The adult height was on average, 2.03 ± 4.4 cm above the mid-parental target height (MPTH).

**Table 4:** Adult height data in GHD males when bone age ≥ 16 years with growth hormone therapy for an average 4.64 ± 1.36 years (n = 12).

Characteristic (by variable name)	N = 12
	Mean ± SD
Adult Height	176.53 ± 5.33
Adult Height SD	0.08 ± 0.69
Mid parental height	173.77± 3.08
Height at Repeat GHST	139.72 ± 13.88
Height SD at Repeat GHST	-1.75 ± 0.73
Height SD before GH treatment	-1.88 ± 0.64
Adult height – Height at Repeat GHST (Delta Height in cm)	36.82 ± 11.35
Adult height - mid parental height	2.10 ± 4.20
Adult height SD – Height SD at GH start	1.86 ± 0.58

p-value^c^: comparing adult height to repeat GST or mid parental target height.

There were too few females in our cohort, and hence the analyses were only limited to male patients. This is consistent with gender disparities, reported previously in idiopathic GHD (28).

## Discussion

Most cases of GHD in pediatric patients are idiopathic, isolated and acquired. All acquired conditions commence with a period during which the diagnosis may not be apparent. However, this may evolve over time (29). We define EGHD as a condition characterized by the progressive decline or insufficient production of growth hormone (GH), especially during the period of pubertal development. It is diagnosed when individuals who were initially deemed GH-sufficient continue to grow inadequately and display signs of inadequate GH secretion, such as decreased growth velocity for age and sex, reduced height SD despite being in puberty, and/or delayed skeletal maturity and are deficient upon GH retesting with GST. We hypothesize that EGHD may be due to evolving hypopituitarism, as the pituitary may be unable to support the compensatory response to normal growth and puberty. EGHD is distinct from ISS and constitutional growth delay due to its unique clinical presentation and responsiveness to GH therapy.

We document here evidence for evolution of GHD in a group of 42 adolescent males who tested sufficient on initial GH stimulation test (GST) but 59% tested deficient upon reevaluation and had an excellent response to GH therapy. Therefore, we considered this an evolving process. Importantly, 32% of these GHD boys were already pubertal when we reevaluated. Clinically, while most characteristics remained similar between boys who tested GHD vs GHS, those with GHD did have a decrease in height Z-score and GV unlike the GHS boys at the repeat GST. Deficient males were treated with GH therapy and demonstrated improvement in height Z-score and growth velocity. All our patients tolerated the treatment well without any adverse effects.

GHD after repeat GST was first reported in patients with organic brain lesions and those who underwent cranial irradiation (18–22, 30). The same phenomenon in the absence of an organic brain lesion or cranial irradiation was first described by Zadik et al. in 1997 (9). This case series highlighted the importance of follow-up of children with unidentified causes of short stature and retesting with GST, especially since they may have been termed as ‘Idiopathic short stature’ otherwise. Our team recently reported two male patients (also currently included in this study) with poor growth and pubertal progression who initially had normal GST but were later diagnosed with EGHD and benefitted from GH therapy (28). GSTs are useful tools in diagnosing GHD, especially in those without obvious causes of GHD such as intracranial tumors or irradiation (9). We acknowledge that several studies of various GH stimulation test protocols in different parts of the world have suggested different GH peak cut offs ranging from 5 to 10 ng/dL (31). But for all practical purposes the current agreed GH cut off in the United States remains to be 10 ng/dL (32). The current published literature on long-acting GH research is also based on the GHD cut off of 10 ng/dl (13, 14). In our patient cohort 36% had a peak ≤ 7 ng/dL on repeat GST; had we waited extended periods, perhaps they may have had lower GH peak levels. Further, to make a diagnosis of GHD in our practice, we also rely on various parameters such as height Z-score, growth velocity, stage of puberty, MPTH, and biochemical markers (such as IGF-1) in addition to the GST. Hence, long-term follow-up and repeat GST along with careful consideration of other biochemical parameters are key to diagnosing patients with EGHD. Treatment may benefit these patients in reaching their expected height potential.

We considered if our patients’ presentation was a result of constitutional delay in growth and puberty (CDGP). However, unlike patients with CDGP, our patients had poor growth velocities in addition to short stature for age or family, while puberty was progressing (33). This is further supported by a study from Binder et al. who compared growth velocities in patients with CDGP and organic GHD from National Cooperative Growth Study (NCGS) registry (n=164) (34). They found that patients with organic GHD had significantly lower height velocity of 3.5 ± 3.2 cm/year compared to height velocity of 5.2 ± 5.4 cm/year in those with CDGP (*p* <.0001) at 14.1 ± 0.4 age. Our prepubertal males with EGHD had a slower growth velocity of 3.34 ± 1.46 cm/yr., comparable to patients with true GHD in the NCGS cohort. Further, growth velocity is typically restored when in puberty for patients with CDGP, but despite 32% of our males being in puberty, their growth velocity was 2.69 ± 0.99 cm/yr. (refer Additional **Table 1**) (33). In a French registry, males treated with GH at a mean chronological age of 13.2 ± 2 years, had a mean height gain of 1.1 ± 0.9 SDS resulting in an average AH of -1.6 SD (boys 165 ± 6 cm) (35). The AH Z-score was 0.4 SD lower than MPTH Z-score and there was a significant concern that the majority of the patients had a constitutional delay of growth and puberty. Hence, although patients with CDGP may receive GH for various concerns, their response is not as robust as those with true GHD, such as our cohort (AH gain of 1.9 SDs) (18, 36).

After one year of treating our patients with GH, the average growth velocity was comparable to the NCGS database, reported as 10.0 ± 1.03 cm/year in their IGHD population at the same age as our population (37). The KIGS database (Pfizer International Growth Databases) reported that children grew at 8.4 ± 2.08 cm/year with GH therapy, similar to our cohort. However, their patients were significantly younger(7.78 ± 2.93 years) compared to our patients (12.04 ± 2.41years) (30). In contrast, patients with ISS after one year of GH treatment were reported to grow at 7.8 ± 2.6 cm/year, slower than our cohort of GHD boys (38, 39). The height Z-score gained) in those with GHD compared to ISS were much higher(0.62 ± 0.33 vs 0.40 ± 0.27, p = 0.03), similar to our patients (0.55 ± 0.22 SDS). Pfäffle et al. compared studies between GHD and ISS patients on GH therapy of which one study reported a height gain of 1.3 SDS over an average of 4.6 years in patients with IGHD while another study reported a gain of only 0.5 SD in patients with ISS over an average of 4.4 years (24). Both the KIGS and NCGS GHD patient cohorts reported mean adult height SDS of -3.1 to 0.2 SDS (40) and -0.7 ± 1.3 (41) respectively, lower than our data after 8.1 years and 4.6 years respectively. Furthermore, Loche et al. studied 15 prepubertal non-GHD children with short stature who were treated with GH and reported their adult heights were not more than their target heights despite having a high mean growth velocity during the first year of therapy (42). A Cochrane review suggested that adult height in patients with ISS is usually lower than their MPTH when compared to individuals with normal stature, hence indicating that it is typically the patients with GHD who are able to reach their genetic potential with GH therapy (43). Our cohort’s response to GH therapy was very similar to multiple reports of patients with GHD, confirming the diagnosis of EGHD All 12 had normal IGF-1 levels measured 3 months or more after therapy and hence did not have repeat GHST. This finding is consistent with previous reports (44).

Our study, additionally supported by adult height data, is a proof-of-concept for the diagnosis of EGHD. We are not aware of previous such reports reported in the literature. In our study we were able to longitudinally follow up almost half of our patients with EGHD to adult height, showing successful therapy with GH without any adverse events. We provide information regarding the characteristics of individuals with EGHD which could be helpful to clinicians in diagnosing and treating this condition.

One of the major limitations of this study is that it is retrospective. In our practice, we do not routinely prime our prepubertal children during GST given the lack of consensus for additional benefit (10, 12, 45, 46). However, eight of our males 32% indeed self-primed as they entered puberty with testosterone reaching pubertal levels and yet tested GHD. We further acknowledge the absence of a control group, as it was not possible to compare outcomes of the treatment with a group of patients who did not receive treatment as they were lost to follow up. We recognize the limitations in reproducibility associated with the Growth Hormone Stimulation Test (GST) and, therefore, integrate the results of GST into a comprehensive clinical and biochemical assessment that is in alignment with the diagnostic criteria for growth hormone deficiency (GHD). We are aware of the lack of consensus regarding the diagnosis of GHD. Several recent publications including multicenter and national studies have used 10 ng/dL as the cut off on GST for GHD diagnosis, as we’ve used in this study (9–12). Lastly, we didn’t explore genetic factors contributing to the development of EGHD, as it is not yet routine to pursue genetic evaluation in individuals with idiopathic, isolated growth hormone deficiency. A recent article by Murray et al. suggested that some causes for short stature in patients with ISS could be explained via genetic testing, which we intend to explore in future studies (47).

In conclusion, we describe a new entity of EGHD among patients evaluated for short stature. We also provide adult height data in the half who have reached adult height that are compatible with results reported for those with IGHD, supporting the diagnosis. Therefore, we strongly recommend that EGHD be considered as part of the differential diagnosis for short stature. Longitudinal careful monitoring and retesting, when necessary, will identify those with EGHD who will benefit from rhGH therapy and will avoid the premature classification of some as ISS.

## Data availability statement

The original contributions presented in the study are included in the article/**Supplementary Material**. Further inquiries can be directed to the corresponding author.

## Ethics statement

The studies involving humans were approved by Icahn School of Medicine at Mount Sinai, Grant Office ID: 19-2771. The studies were conducted in accordance with the local legislation and institutional requirements. Written informed consent for participation in this study was provided by the participants’ legal guardians/next of kin.

## Author contributions

SC: Writing – review & editing, Writing – original draft. SS: Writing – original draft, Formal analysis, Data curation, Conceptualization. JS: Writing – review & editing, Data curation. AK: Writing – review & editing, Formal analysis. CM: Writing – review & editing, Visualization, Data curation. MS: Writing – review & editing. RR: Writing – review & editing, Writing – original draft.
