# Supplementary material for: Evolving growth hormone deficiency: proof of concept
Source: Front Endocrinol (Lausanne). 2024 May 1;15:1398171. doi: 10.3389/fendo.2024.1398171 (PMC11095394; doi:10.3389/fendo.2024.1398171)
Supplement: Supplementary file 1 [file Table_1.docx]

**Supplemental Table 1:**

Table 5. Pubertal vs Non-pubertal GHD males’ characteristics over time:

|  | **Pubertal**  **(N=8)** | **Non-pubertal (N=18)** | **p-value** |
| --- | --- | --- | --- |
|  | **Mean (SD)** | **Mean (SD)** |  |
| **At repeat GST** | | | |
| Peak GH (ng/mL) | 6.73 (2.53) | 7.91 (1.92) | 0.1431 |
| Height SD score | -1.46 (0.75) | -1.96 (0.54) | 0.0639 |
| Testosterone (ng/dL) | 218.71 (281.21) | 39.53 (95.85) | **0.0070** |
| Testicular volume (mL) | 10.86 (5.93) | 3.00 (1.51) | **0.0016** |
| Growth velocity (cm/year) | 3.34 (1.46) | 2.87 (1.29) | 0.6443 |
| Growth velocity SDS | -1.01 (1.16) | -1.25 (1.63) | 0.2717 |
| Bone age - Chronological age | -1.00 (1.04) | -1.01 (0.68) | 0.2387 |
| IGF-1 Z-score | -1.27 (1.19) | -1.01 (0.68) | 0.5906 |
| **At 1 year after GH therapy** | | | |
| Height SD before rhGH | -1.68 (0.70) | -2.00 (0.55) | 0.1849 |
| Height SD score | -1.21 (0.54) | -1.41 (0.61) | 0.5627 |
| Growth velocity (cm/year) | 8.84 (2.43) | 9.06 (1.11) | 0.7421 |
| **At most recent visit** | | | |
| rhGH duration | 2.20 (1.80) | 2.35 (3.61) | 0.6383 |
| Height SD score | 0.04 (1.59) | -0.90 (0.71) | 0.1087 |
| Testicular volume (mL) | 18.00 (7.56) | 12.76 (8.90) | 0.1863 |
| Growth velocity (cm/year) | 4.33 (3.13) | 5.27 (3.18) | 0.4929 |
| Growth velocity SDS | 0.94 (1.32) | 0.78 (1.80) | 0.6319 |
| Bone age - Chronological age | -1.96 (1.27) | -0.63 (1.69) | 0.1730 |
| IGF-1 Z-score | 0.92 (2.18) | 1.60 (1.36) | 0.4603 |
